# Reviewed article: Batista JFC, Santos MAA, Lima SO. Trend of epidemiological indicators and factors associated with tuberculosis treatment dropout and death among street people in Brazil: an ecological and cross-sectional study, 2014-2022. Epidemiol Serv Saude. 2025:34;20240273

**DOI:** 10.1590/S2237-96222024v34e20240273.b

**Published:** 2025-04-14

**Authors:** Daniela de Almeida Pereira

**Affiliations:** Universidade Federal de Vicosa

**Table:** 

Rating	Excellent	Good	Average	Below average	Poor
Interest		✓			
Quality		✓			
Originality		✓			
Overall		✓			

**Table:** 

Questionnaire	Yes	No	Not applicable
Does the manuscript contain new and significant information to justify publication?	✓		
Does the Abstract (Summary) clearly and accurately describe the content of the article?		✓	
Is the problem significant and concisely stated?	✓		
Are the methods described comprehensively?		✓	
Are the interpretations and conclusions justified by the results?	✓		
Is adequate reference made to other work in the field?	✓		

## Manuscript Structure:

Length of article is: Adequate

Number of tables is: Adequate

Number of figures is: Adequate

Please state any conflict(s) of interest that you have in relation to the review of this paper (state “none” if this is not applicable): None

## Comments

Parabéns pelo trabalho realizado. Os moradores de rua são pessoas que vivem em situação precária que contribuem para o surgimento de diversas doenças, dentre elas a tuberculose. Para melhor prognóstico, essa doença precisa de um diagnóstico precoce e monitoramento de todo tratamento, o que não é fácil, uma vez que a população de rua, em geral, não se fixa em um local e alguns resistem aos cuidados e tratamento. Visando contribuir com o estudo para melhor compreensão do leitor, seguem algumas dúvidas e/ou sugestões:

O texto precisa ser revisado para garantir clareza, precisão e objetividade na linguagem e as ideias devem ser apresentadas de forma organizada e lógica. Por exemplo: página 2, linha 22: “Foram 21.904 casos. A tendência da cura foi de redução no Brasil e macrorregiões, enquanto a incidência e mortalidade reduziu e na região Sudeste”, parece que a frase faltou complementação. No objetivo “Analisar a tendência temporal dos indicadores epidemiológicos...”, e no delineamento da metodologia: “...subdividido em análise temporal dos indicadores da tuberculose...”, nesse início não fica claro quais indicadores, somente em variáveis (página 3, linha 44) são citados os indicadores, sugiro que sejam citados no trecho do delineamento para compreensão do leitor. A organização dos subtítulos da metodologia ficou um pouco confusa, para melhor compreensão sugiro que a Fonte dos dados seja colocada antes de Participantes e após o Contexto. E a justificativa para seleção a partir de 2014 seja descrita dentro de contexto. De acordo com os objetivos, o estudo analisou 2 variáveis dependentes (abandono de tratamento e óbito). No detalhamento das variáveis (página 4, linha 40) entende-se que é apenas abandono de tratamento, sugiro a inclusão de óbito. No subtítulo Dados individuados (página 5, linha 3) passa o entendimento de que serão analisados dados individuais, mas não é o caso do estudo, sugiro a retirada ou substituição para “Análise das variáveis dependentes”. Nos resultados, página 6, linha 34, é descrita que a maioria da população reside na macro Centro-Oeste, porém de acordo com a tabela 2 seria a região Sudeste. Na linha 42, página 6 é citada tabela 5, porém ela não acompanha o trabalho. Na página 7, linha 46, em discussão, é citada redução na região Sudeste, e depois cita uma referência sobre o Paraná como evidência contrária, porém acho que ficou confuso, uma vez que o Paraná pertence ao Sul e no Sul houve crescimento, não é isso?

